# Major Traumatic and Severe Thermal Injuries Lead to Immediate and Persistent Elevations in Circulating Concentrations of Resistin That Are Associated with Poor Clinical Outcomes and Impaired Innate Immune Responses

**DOI:** 10.3390/biom16030443

**Published:** 2026-03-16

**Authors:** Emily Horner, Kirsty C. McGee, Sebastian Tullie, David N. Naumann, Animesh Acharjee, Thomas Lissillour, Ali Asiri, Janice M. S. Ng, Jack Sullivan, Amanda V. Sardeli, Paul Harrison, Antonio Belli, Naiem S. Moiemen, Janet M. Lord, Jon Hazeldine

**Affiliations:** 1 Department of Inflammation and Ageing, School of Infection, Inflammation and Immunology, College of Medicine and Health, University of Birmingham, Birmingham B15 2TT, UK; 2 National Institute for Health and Care Research (NIHR) Birmingham Biomedical Research Centre, Birmingham B15 2TH, UK; 3 University Hospitals Birmingham NHS Foundation Trust, Queen Elizabeth Hospital Birmingham, Birmingham, B15 2GW, UK; 4 Academic Department of Military Surgery and Trauma, Royal Centre for Defence Medicine, Queen Elizabeth Hospital Birmingham, Birmingham B15 2GW, UK; 5 Institute of Cancer and Genomic Sciences, University of Birmingham, Birmingham B15 2TT, UK; 6 The Scar Free Foundation Centre for Conflict Wound Research, Queen Elizabeth Hospital Birmingham, Birmingham B15 2GW, UK; 7 National Institute for Health Research Surgical Reconstruction and Microbiology Research Centre, Queen Elizabeth Hospital Birmingham, Birmingham B15 2GW, UK; 8 MRC-Versus Arthritis Centre for Musculoskeletal Ageing Research, University of Birmingham, Birmingham B15 2TT, UK

**Keywords:** burns, critical care, immune suppression, resistin, traumatic injury

## Abstract

Major trauma induces innate immune suppression, yet the underlying mechanisms are poorly understood. Resistin is an immunosuppressive molecule that is systemically elevated post-injury. However, its role in trauma-induced immune dysfunction and clinical outcomes is poorly defined. Here, we acquired blood samples from 147 adult trauma patients (≤1, 4–12, 48–72 h post-injury) and 95 burns patients (days 1, 3, 7, 14, 28 post-burn). We measured plasma resistin concentrations, studied resistin gene expression in peripheral blood mononuclear cells (PBMCs) and neutrophils, and measured resistin production by lipopolysaccharide (LPS)-challenged whole blood leukocytes. To identify potential novel triggers of resistin secretion by immune cells, we examined the effect that stimulation with mitochondrial-derived damage-associated molecular patterns (mtDAMPs) had on resistin production by neutrophils isolated from healthy donors. We also treated neutrophils, from healthy donors, and THP-1 cells with resistin prior to stimulation with Phorbol 12-myristate-13-acetate (PMA) or LPS to study its effects on reactive oxygen species (ROS) and cytokine production, respectively. Injured patients presented with significantly elevated circulating resistin concentrations and increased resistin gene expression in PBMCs and neutrophils. LPS and mtDAMP stimulation promoted resistin secretion by whole blood leukocytes and neutrophils. Plasma resistin concentrations were negatively associated with PMA-induced ROS generation by neutrophils, and LPS-induced cytokine production by monocytes. Resistin-treated THP-1 cells and neutrophils exhibited impaired functional responses upon secondary stimulation with LPS or PMA, respectively. Trauma patients who developed multiple organ dysfunction syndrome (MODS) presented with significantly elevated resistin concentrations, which at 48–72 h post-injury showed good performance as a predictor of post-traumatic MODS (AUROC, 0.796). Hyperresistinemia is an immediate and persistent feature of the inflammatory response to injury that may contribute to the development of innate immune dysfunction.

## 1. Background

Characterised by concurrent episodes of immune activation and suppression, major traumatic and severe thermal injuries result in a state of systemic immune dysregulation [1,2,3,4]. With the aim of restoring immune homeostasis, a compensatory anti-inflammatory response syndrome (CARS) is triggered within minutes of a sterile insult and persists into the acute injury phase, where it counteracts the pro-inflammatory systemic inflammatory response syndrome (SIRS) [5]. Evident in both the innate and adaptive arms of the immune system, defining features of the CARS response include: (i) impaired reactive oxygen species (ROS) generation by neutrophils, (ii) reduced ex vivo production of pro-inflammatory cytokines by lipopolysaccharide (LPS)-challenged monocytes, and (iii) decreased expression of the MHC Class II molecule HLA-DR on the surface of monocytes [2,4,5,6,7,8,9]. When dysregulated, a CARS response of increased magnitude and/or duration increases the susceptibility of injured patients to the development of such secondary complications as nosocomial infections, sepsis, and multiple organ dysfunction syndrome (MODS) [10,11,12]. Notably, despite this clinical relevance, the factors that promote injury-induced immune suppression are poorly understood.

Belonging to the found in inflammatory zones (FIZZ) protein family, resistin is a 12.5 kDa cysteine-rich protein. In contrast to rodents, where resistin is produced primarily by adipocytes, immune cells are considered the predominant source of resistin in humans, with neutrophils, circulating mononuclear cells, and macrophages all reported to express and secrete resistin upon inflammatory challenge [13,14,15,16]. A protein with pro-inflammatory properties, resistin is a potent immunomodulatory molecule, with a significant number of studies highlighting its ability to suppress neutrophil and monocyte anti-microbial functions. For example, treatment of human mononuclear cells with resistin prior to LPS challenge was shown to reduce tumour necrosis factor-alpha (TNF-α) and interleukin (IL)-6 production [17,18], whilst resistin-treated neutrophils exhibited impaired chemotactic responses, ROS generation, and bactericidal activity upon secondary stimulation [19,20,21,22]. In line with these findings, culturing neutrophils in serum isolated from patients with hyperresistinemia has been shown to inhibit their chemotactic activity and generation of ROS [15,22], with these functional impairments restored when resistin levels in serum were reduced via hemoadsorption [22]. Interestingly, these resistin-induced changes in innate immune cell behaviour mirror the dysfunctional responses exhibited by neutrophils and monocytes isolated from patients that have sustained major traumatic and thermal injuries [1,2,3,4,23,24].

Compared to levels detected in healthy volunteers, elevated circulating concentrations of resistin have been measured in burns and traumatically brain-injured patients [25,26,27,28,29,30,31]. Significantly raised serum and plasma concentrations of resistin were recorded during the acute (6–24 h) and subacute (days 2–10) phases, where they correlated positively with inflammatory markers (e.g., C Reactive Protein, plasminogen activator inhibitor-1, monocyte chemoattractant protein, TNF-α, IL-6, IL-8) [25,26,27,28,29]. Moreover, results from prospective-based studies have linked injury-induced hyperresistinemia to poor clinical outcomes, with non-survivors and patients with MODS presenting with significantly elevated levels of resistin [25,28,29,32], which in a cohort of traumatically brain-injured patients was shown to be an independent predictor of 1-month mortality [28].

To date, no study to our knowledge has examined whether a relationship exists between resistin and trauma-induced immune dysregulation. Whilst the results of the aforementioned in vitro studies would suggest that hyperresistinemia could promote a prolonged and/or exacerbated CARS response by impairing innate immune responses [17,18,19,20,21,22], it should be noted that the concentrations of resistin used in these experiments (100–1000 ng/mL) far exceed those measured in the circulation of burns and traumatically brain-injured patients (1–35 ng/mL) [17,18,19,21,25,26,27,28]. This therefore raises the question as to whether resistin, at the levels recorded post-injury, can modulate the anti-microbial responses of innate immune cells. Furthermore, researchers have highlighted the fact that the mechanism(s) leading to the elevation of resistin and its association with clinical outcomes in the setting of critical care remain relatively unexplored [33]. Thus, it has been suggested that a greater emphasis needs to be placed on addressing this knowledge gap if we are to prevent and/or treat the potentially debilitating long-term consequences reported by major trauma survivors, such as chronic critical illness [34], a condition for which systemic immunosuppression is a defining feature [35].

With the aim of investigating whether a relationship exists between resistin and post-injury systemic immune modulation, we have performed a comprehensive analysis of peripheral blood samples acquired from 242 major traumatic and severe thermally-injured patients. Analysing samples collected across the ultra-early (≤1-h), immediate (4–12 h), acute (days 2–7), and subacute (days 14–28) injury phases, we have examined whether associations exist between circulating resistin concentrations, markers of systemic inflammation and immune function, and determined whether measurements of resistin can distinguish between patients with differing clinical outcomes, specifically the development of MODS following major trauma. In addition, we have examined the potential cellular sources of resistin, as well as the triggers and signalling pathways involved in injury-induced resistin secretion by leukocytes. Finally, we have investigated whether resistin, at concentrations measured in injured patients, can suppress the anti-microbial activities of neutrophils and induce endotoxin tolerance in THP-1 cells.

## 2. Methods

### 2.1. Study Design and Setting

This study presents data generated from the analysis of blood samples collected from patients enrolled into two independent prospective observational cohort studies based at a single Major Trauma Centre in the United Kingdom (University Hospitals Birmingham NHS Foundation Trust (UHBFT), Birmingham). The Scientific Investigation of the Biological Pathways Following Thermal Injury-2 (SIFTI-2) study recruited adult (≥16 years) burns patients that were admitted to the West Midlands Regional Burns Centre (WMRBC) within 24 h of sustaining a total body surface area (TBSA) burn ≥ 15%. Ethical approval for the study (trial registration number: NCT04693442) was granted on 7 June 2016 by the West Midlands, Coventry and Warwickshire Research Ethics Committee (REC reference:16/WM/0217). The Brain Biomarkers after Trauma Study (BBATS), recruited adult (≥18 years) trauma patients within 1 h of sustaining a traumatic injury with an injury severity score (ISS) ≥ 8. The North Wales Research Ethics Committee-West granted ethical approval for the BBATS study on 3 April 2014 (REC reference:13/WA/0399, Protocol Number: RG_13-164).

### 2.2. Patient Cohorts

#### 2.2.1. Burns

Data derived from serial peripheral blood samples acquired from 95 adult burns patients enrolled into the SIFTI-2 study after sustaining a TBSA burn ≥ 15% between November 2016 and December 2023 are presented in this manuscript. Information relating to the design of the SIFTI-2 study, and the process of patient consent and recruitment are outlined in the study protocol [36]. The number of patient samples analysed differ across timepoints due to patient mortality, refusal of additional blood sampling, hospital discharge, or inadequate sampling volumes for processing.

#### 2.2.2. Trauma

Data generated from the analysis of serial blood samples obtained from 147 subjects enrolled into the BBATS study between May 2014 and August 2018 are presented here. On a 24/7 working pattern, pre-hospital critical care paramedics obtained peripheral blood samples from adult trauma patients (≥18 years; suspected ISS ≥ 8) within 1 h of injury (defined as the time of phone call to emergency services). Information relating to the processes of patient consent, study enrolment, and inclusion/exclusion criteria have been described previously [2]. The number of patient samples analysed differ between timepoints due to patient mortality, refusal of additional blood sampling, hospital discharge, or inadequate sampling volumes for processing.

#### 2.2.3. Healthy Controls (HCs)

In total, 45 HCs (mean age, 33 years; range 20–65 years; 30 males, 15 females) were recruited in accordance with the ethical approval provided by the University of Birmingham Research Ethics Committee (Ref: ERN_12-1184). HCs were defined as individuals who were not taking any regular medication for a diagnosed illness and who had not experienced an infectious episode in the two weeks prior to sampling.

### 2.3. Clinical Data Collection

Demographic data and injury details for burns and trauma patients were obtained prospectively from electronic and physical medical records. Data collected included patient age, sex, mechanism of injury, body mass index (BMI), severity of injury (ISS and Glasgow Coma Scale (GCS) score), time of injury, percent TBSA, percent TBSA full thickness, Baux score, revised Baux score, abbreviated burn severity index (ABSI), and sequential organ failure assessment (SOFA) score.

### 2.4. Patient Outcomes

Clinical data relating to patient mortality and both Intensive Care Unit (ICU) and hospital-free days (calculated as 30 minus the number of days the patient stayed in ICU and hospital, respectively) were extracted from electronic records. Patients who died in the hospital or ICU setting within 30 days of admission were assigned a score of 0. In our trauma cohort, the development of MODS was defined as a SOFA score of 6 or more, on two consecutive days, at least 48 h post-hospital admission [37].

### 2.5. Blood Sampling

For trauma patients, data were obtained from the analysis of blood samples collected into BD Vacutainers^®^ (BD Biosciences, Berkshire, UK) containing lithium heparin, a 1/10 volume of 3.2% trisodium citrate or z-serum clotting activator, at three post-injury timepoints pre-hospital (≤1 h), 4–12, and 48–72 h. During transport to QEHB, samples were stored at room temperature (RT), where, upon arrival, they were stored at 4 °C. Specimens were collected for analysis within 1 h of arrival at QEHB. Data from burns patients was acquired via the analysis of peripheral blood samples collected into BD Vacutainers^®^ containing a 1/10 volume of 3.2% trisodium citrate or z-serum clotting activator at the following post-injury timepoints: Days 1, 3, 7, 14 and 28. Once acquired, samples were delivered to the hospital-based laboratory within 1 h and processed immediately.

### 2.6. Preparation of Platelet-Free Plasma (PFP), Serum, and Mitochondrial-Derived Damage-Associated Molecular Patterns (mtDAMPs)

Resistin concentrations were measured in PFP samples. PFP was prepared from blood collected into vacutainers containing a 1/10 volume of 3.2% trisodium citrate and stored at −80 °C prior to analysis as described previously [3]. Serum was prepared from peripheral blood samples collected into vacutainers containing z-serum clotting activator. Following a 30 min incubation at RT, blood samples were subjected to centrifugation at 1620× *g* for 10 min at RT. Post-centrifugation, serum aliquots were collected and stored at −80 °C prior to analysis. PFP and serum samples were stored in single-use aliquots to ensure no freeze-thaw cycles prior to inclusion in experimental assays.

MtDAMPs were prepared from the K562 cell line purchased from the American Type Culture Collection (ATCC^®^, Teddington, Middlesex, UK) as described previously [38]. Protein concentrations were determined by spectrophotometry, with samples stored in single-use aliquots at −80 °C prior to use.

### 2.7. Quantification of Circulating Resistin Concentrations

Resistin concentrations were measured in PFP samples using a commercially available Duo Set ELISA kit as per manufacturer’s instructions (R and D Systems, Oxford, UK).

### 2.8. Quantification of Cytokines, Chemokines, and Mitochondrial Encoded NADH Dehydrogenase 6 (ND6)

Concentrations of granulocyte colony-stimulating factor (G-CSF), monocyte chemoattractant protein (MCP-1), TNF-α, IL-6, IL-8, and/or IL-10 were measured in serum samples or cell-free culture supernatants using a magnetic bead-based multiplex immunoassay kit as described in manufacturer’s guidelines (Bio-Rad, Hertfordshire, UK). Serum concentrations of ND6 were measured using a commercially available ELISA as per manufacturer’s instructions (MyBioSource, San Diego, CA, USA).

### 2.9. LPS Stimulation of Whole Blood Leukocytes

400 µL aliquots of heparinised whole blood were stimulated for 4 h (37 °C/5%CO_2_) with 10 ng/mL LPS from *Escherichia coli* (serotype 0111:B4; Merck Life Science UK Limited, Dorset, UK) or vehicle control. Post-stimulation, samples were subjected to centrifugation for 8 min at 461× *g* at 4 °C. Supernatants were subsequently collected and stored at −80 °C prior to analysis. Resistin concentrations were quantified in accordance with instructions detailed in the manufacturer’s instructions of a commercially available Duo Set ELISA Kit (R and D Systems).

### 2.10. Neutrophil ROS Production in Whole Blood

Following manufacturers guidelines, a commercially available PhagoBURST assay (BD Biosciences, Oxford, UK) was used to measure ROS production by neutrophils following stimulation of 100 μL aliquots of heparinised whole blood with 1.62 μM PMA. Samples were analysed on an Accuri C6 flow cytometer, where 10,000 neutrophils were gated and their mean fluorescence intensity (MFI) values recorded.

### 2.11. HLA-DR Staining

100 μL aliquots of heparinised whole blood were stained on ice for 30 min with 1 μg/mL mouse anti-human CD14-FITC (clone TUK4; Dako, Cambridgeshire, UK) and 0.07 μg/mL HLA-DR-PE (clone LN3; ThermoFisher Scientific, Chesire, UK) or its concentration matched isotype control (Mouse IgG2B-PE, BioLegend, Cambridge, UK). Post-incubation, red blood cells were lysed (BD PharmLyse, BD Biosciences) and samples fixed at room temperature (RT) for 20 min with 50 μL of fixation medium (Life Technologies, Paisley, UK). Samples were washed once in phosphate-buffered saline (PBS; 250× *g*, 5 min, 4 °C), resuspended in 200 µL PBS and analysed on a CyAn_ADP_ bench top cytometer. HLA-DR expression was recorded as median fluorescence intensity (MedFI) values.

### 2.12. Cell Culture and Isolation of Neutrophils and Peripheral Blood Mononuclear Cells (PBMCs)

PBMCs were extracted from heparinised anti-coagulated whole blood samples using Ficoll-Paque PLUS media (Cytiva, Sheffield, UK) and density gradient centrifugation or from citrated anti-coagulated whole blood samples by Percoll density gradient centrifugation (Merck Life Science UK Limited). Post-isolation, PBMC numbers were calculated using a Sysmex NX-1000 haematology analyser (Sysmex UK, Milton Keynes, UK). Neutrophils were isolated from heparinised or citrated anti-coagulated blood samples by Percoll density gradient centrifugation. Determined via use of the Sysmex XN-1000 haematology analyser, the purity of neutrophil preparations was routinely ≥98%.

Human monocytic THP-1 cells, purchased from the ATCC^®^ (Manassas, VA, USA), were cultured at 37 °C/5%CO_2_ in complete media (CM), which comprised of RPMI-1640 media supplemented with 2 mM L-glutamine, 100 U/mL penicillin, 100 µg/mL streptomycin (GPS) and 10% heat-inactivated fetal calf serum (Merck Life Science UK Limited).

### 2.13. LPS Stimulation of Isolated PBMCs

Purified PBMCs (1 × 10^6^) in a total volume of 500 µL CM were dispensed into 24 well plates and stimulated for 4 h (37 °C/5%CO_2_) with 10 ng/mL LPS (Merck Life Science UK Limited) or vehicle control. Post-challenge, supernatants were collected and centrifuged at 1500× *g* for 2 min at 4 °C. Cell-free supernatants were harvested and stored at −80 °C until analysed. Resistin concentrations in supernatants were quantified according to the protocol described in the manufacturer’s instructions of a commercially available Duo Set ELISA Kit (R and D Systems).

### 2.14. Stimulation of Purified Neutrophils

For experiments with unprimed cells, purified neutrophils (1 × 10^6^) in a total volume of 200 µL CM were dispensed into 96 well round bottomed plates and stimulated for 2 h (37 °C/5%CO_2_) with either 10 ng/mL TNF-α (Merck Life Science UK Limited), 10 ng/mL granulocyte-macrophage colony-stimulating factor (GM-CSF; Merck Life Science UK Limited), 100 ng/mL IL-8 (R and D Systems), 100 ng/mL LPS (Merck Life Science UK Limited), 100 µg/mL mtDAMPs or vehicle control. For priming experiments, purified neutrophils (1 × 10^6^) were treated for 30 min with 10 ng/mL GM-CSF (37 °C/5%CO_2_) prior to a 30 min stimulation with 100 µg/mL mtDAMPs. In these experiments, neutrophils treated with GM-CSF (10 ng/mL) or mtDAMPs (100 µg/mL) alone served as controls. To investigate the role of formyl peptide signalling in mtDAMP-induced resistin secretion, purified neutrophils (1 × 10^6^) were treated for 1 h (37 °C/5%CO_2_) with 1 µM of the formyl peptide receptor-1 (FPR-1) inhibitor Cyclosporin H (CsH, Abcam, Cambridge, UK) or vehicle control prior to a 30 min priming with 10 ng/mL GM-CSF and 30 min stimulation with 100 µg/mL mtDAMPs. Post-treatment, supernatants were collected via centrifugation (1500× *g*, 2 min, 4 °C) and stored at −80 °C prior to analysis. Resistin concentrations in supernatants were calculated using the protocol outlined in the manufacturer’s instructions of a commercially available Duo Set ELISA Kit (R and D Systems).

### 2.15. Real-Time Polymerase Chain Reaction (RT-PCR)

PBMCs and neutrophils (5 × 10^6^) isolated from citrated anti-coagulated blood were re-suspended in 1 mL of TRIzol reagent (Life Technologies, Cheshire, UK) and stored at −80 °C prior to RNA extraction using the protocol outlined in manufacturer’s instructions. Extracted RNA was resuspended in 20 µL RNase-free water and heated at 55 °C for 10 min, after which its concentration was determined using a NanoDrop 2000 (ThermoFisher Scientific). Typical A260/A280 ratios for isolated RNA were between 1.8 and 2.0, which we considered suitable for analysis in RT-PCR assays.

For RT-PCR, mRNA levels of resistin were determined, relative to the housekeeping gene 18S, using the iTaq^™^ Universal SYBR^®^ Green One-step kit mastermix (Bio-Rad) and gene-specific primers; resistin forward primer, 5′CTGTTGGTGTCTAGCAAGACC3′; resistin reverse primer 5′CCAATGCTGCTTATTGCCCTAAA3′; 18S forward primer, 5′GTAACCCGTTGAACCCCATT3′; 18S reverse primer, 5′CCATCCAATCGGTAGTAGCG3′. Reactions were performed in triplicate in a total volume of 5 µL that contained 5 ng RNA. A non-template control comprising of iTaq^™^ Universal SYBR^®^ Green One-step mastermix and gene-specific primers was performed in each RT-PCR to check for contamination of PCR reagents. RT-PCRs were ran on a Bio-Rad sfx cycler (Bio-Rad), with data extracted using Bio-Rad CFX manager software (Bio-Rad) and analysed via the 2^−ΔΔCt^ method.

### 2.16. In Vitro Resistin Treatment

Neutrophils (1 × 10^6^/mL) in hanks balanced salt solution supplemented with calcium and magnesium (HBSS^+/+^) were treated for 1 h (37 °C/5%CO_2_) with 150 ng/mL recombinant human resistin protein (sourced from HEK293 cells; AssayGenie, Dublin, Ireland) or vehicle control. Post-treatment, 100 μL aliquots were dispensed into wells of a 96-well white-bottomed flat plate (BD Biosciences), pre-coated with PBS/2% BSA, that contained 25 μL of lucigenin (final concentration 200 μM; Merck Life Science UK Limited) and 50 μL HBSS^+/+^. Neutrophils were subsequently challenged with 25 nM PMA or vehicle control, and ROS production measured at 1 min intervals for a total time of 180 min using a Berthold Centro LB 960 luminometer (Berthold Technologies, Hertfordshire, UK). Samples were analysed in quadruplicate, with ROS production recorded as relative light units and calculated as area under the curve (AUC).

THP-1 cells (1 × 10^6^ in CM) were cultured for 24 h (37 °C/5%CO_2_) in the presence of either 150 ng/mL recombinant human resistin or vehicle control. Post-incubation, cells were stimulated for 4 h with 1 µg/mL LPS at 37 °C/5%CO_2_, after which supernatants were collected and centrifuged at 1500× *g* for 2 min at 4 °C. Cell-free supernatants were harvested and stored at −80 °C prior to the analysis of TNF-α concentrations using a commercially available Duo Set ELISA Kit (R and D Systems).

### 2.17. Statistical Analyses

Data distribution was determined using the Kolmogorov–Smirnov or Shapiro–Wilk normality tests. Normally distributed data were analysed via a paired student *t*-test or a repeated measures one-way ANOVA with Tukey’s multiple comparison post hoc test. Non-normally distributed data were analysed using a Mann–Whitney U test, a Wilcoxon matched-pairs signed rank test, and for multiple comparisons, a Kruskal–Wallis test with Dunn’s multiple comparison post hoc test. Relationships between continuous variables were assessed using a Spearman’s or Pearson’s correlation. Benjamini–Hochberg corrections (pFDR < 0.05) were performed to account for multiple testing. For comparisons of continuous variables between patients who did or did not develop MODS, Mann–Whitney U tests or independent samples *t*-tests were performed, whilst Chi-squared tests were conducted to compare categorical variables. Area under the receiver-operating curve (AUROC) analyses were performed to assess the ability of resistin concentrations, measured <1, 4–12, and 48–72 h post-injury, to discriminate between patients who did or did not develop post-traumatic MODS. For all statistical tests, the threshold for statistical significance was set at *p* ≤ 0.05. Statistical analyses were performed using GraphPad PRISM 10 software (GraphPad Software Ltd., San Diego, CA, USA) and SPSS statistics (Version 30.0.0.0, IBM). For violin plots, individual samples are depicted by dots, with dashed lines representing quartiles and the solid line presenting the median value. For histograms, data are presented as mean ± standard error of the mean.

## 3. Results

### 3.1. Patient Demographics

Blood samples acquired from 147 traumatically-injured patients (126 males, 21 females) were analysed in this study (Table 1). Patients had a mean age of 42 years (range 18–95 years) and a mean ISS of 25 (range 9–66). Road traffic collisions were the predominant mechanism of injury (53%), with a mean time to pre-hospital blood sampling of 42 min post-injury (range 13–60 min).

Data derived from peripheral blood samples obtained from 95 adult thermally-injured patients (75 males, 20 females) who presented with a mean TBSA burn of 36% (range 15–85%) are presented in this study (Table 1). Patients had a mean age of 47 years (range 16–83 years), with flame burn the predominant mechanism of injury (83%).

### 3.2. Major Traumatic and Severe Thermal Injury Results in Rapid and Persistent Elevations in Plasma Concentrations of Resistin

**Figure 1:**
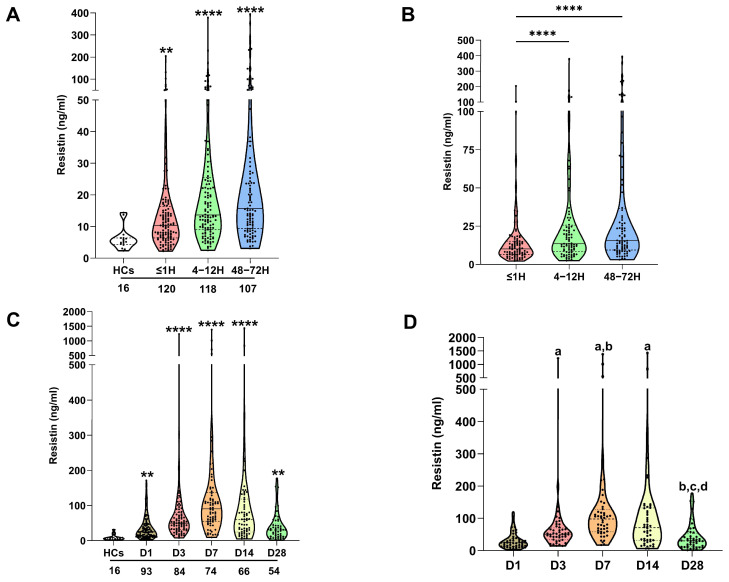
Traumatic and thermal injury results in elevated circulating concentrations of resistin. (**A**) Comparison of resistin concentrations in platelet-free plasma collected from healthy controls (HCs) and major trauma patients at three post-injury timepoints (≤1, 4–12, and 48–72 h). ** *p* < 0.005, **** *p* < 0.0001 vs. HCs. The number of samples analysed are stated below each study time-point. (**B**) Repeated measures analyses comparing resistin concentrations in platelet-free plasma obtained from major trauma patients (*n* = 88) at three post-injury timepoints (≤1, 4–12, and 48–72 h). **** *p* < 0.0001 vs. ≤1 h. (**C**) Comparison of resistin concentrations in platelet-free plasma collected from healthy controls (HCs) and thermally-injured patients at five post-burn timepoints (days 1, 3, 7, 14, and 28). ** *p* < 0.005, **** *p* < 0.0001 vs. HCs. The number of samples analysed are stated below each study time-point. (**D**) Repeated measures analyses comparing resistin concentrations in platelet-free plasma obtained from burns patients (*n* = 48) at days 1, 3, 7, 14, and 28 post-injury. ^a^ *p* < 0.0005 vs. D1, ^b^ *p* < 0.01 vs. D3, ^c^ *p* < 0.0001 vs. D7, ^d^ *p* < 0.0001 vs. D14.

Compared to HCs, trauma patients presented at all post-injury study timepoints (≤1 h, 4–12, and 48–72 h) with significantly elevated plasma concentrations of resistin (Figure 1A). No relationship was observed between patient age and resistin levels (Supplementary Table S1), and resistin concentrations were comparable between male and female subjects (Supplementary Figure S1A). For patients from whom samples were acquired at all study time-points, repeated measures analyses revealed resistin concentrations were significantly elevated 4–12 and 48–72 h post-injury when compared to samples acquired ≤1-h post-trauma (Figure 1B).

In a cohort of 95 adult major burns patients, significantly higher concentrations of resistin were detected in plasma samples obtained at days 1, 3, 7, 14, and 28 post-injury when compared to levels recorded in HCs (Figure 1C). At day 28 post-burn, a positive association was detected between patient age and plasma resistin concentrations (Supplementary Table S1). On day 7 post-burn, female patients presented with significantly elevated resistin levels when compared to their male counterparts (Supplementary Figure S1B). At no study timepoint was a relationship observed between plasma resistin concentrations and BMI scores (Supplementary Table S1). Repeated measures analyses found resistin concentrations were significantly elevated on days 3, 7, and 14 post-burn when compared to the levels measured on the day of injury (Figure 1D). At day 28 post-burn, patients presented with resistin concentrations that were significantly lower than those recorded on days 3, 7, and 14 post-injury and comparable to those measured on day 1 of injury (Figure 1D).

### 3.3. Elevated Resistin Concentrations Are Associated with the Development of MODS Following Major Traumatic Injury

**Figure 2:**
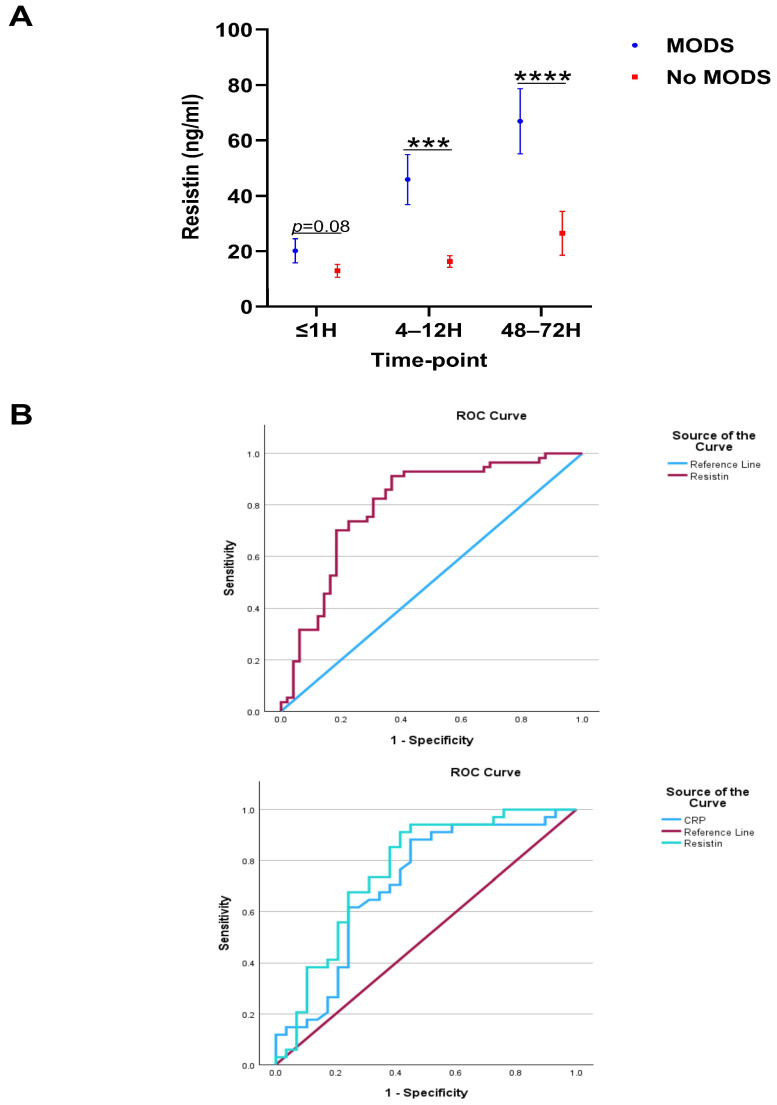
Elevated concentrations of plasma resistin are associated with poor clinical outcome following major and traumatic injury. (**A**) Comparison of resistin concentrations measured in platelet-free plasma samples acquired at three post-injury timepoints (≤1, 4–12, and 48–72 h) between patients who did or did not develop post-traumatic multiple organ dysfunction syndrome (MODS; ≤1, MODS = 52, No MODS = 57; 4–12, MODS = 54, No MODS = 55 and 48–72 MODS = 57, No MODS = 49). *** *p* < 0.0005, **** *p* < 0.0001. (**B**) Top Panel: ROC curve analysis examining the accuracy of plasma resistin concentrations, measured 48–72 h post-injury, at distinguishing between patients who did (*n* = 57) or did not (*n* = 49) develop MODS. Bottom Panel: ROC curve analysis examining the accuracy of plasma resistin and C reactive protein (CRP) concentrations, measured 48–72 h post-injury, at distinguishing between patients who did (*n* = 34) or did not (*n* = 29) develop MODS.

With incidence rates as high as 55%, MODS is a common secondary complication amongst traumatically-injured patients and is associated with poor clinical outcomes [39]. A comparison of plasma resistin concentrations between trauma patients who did or did not develop MODS (Supplementary Table S2) revealed that MODS patients presented at the 4–12 and 48–72 h sampling timepoints with significantly elevated resistin levels (Figure 2A). Subsequent ROC curve analysis, performed to assess the accuracy of resistin as a potential prognostic factor for the development of post-traumatic MODS, revealed greatest discriminatory power at the 48–72 h sampling timepoint where, an AUC value of 0.796 (95%CI, 0.707–0.885) was calculated (Figure 2B). In comparison, AUC values of 0.597 (95%CI, 0.490–0.705) and 0.686 (95%CI, 0.585–0.787) were generated from the levels of resistin measured in plasma acquired ≤1 h and 4–12 h post-injury, respectively. In a subgroup of patients (*n* = 63), measurements of plasma resistin and C-reactive protein (CRP) concentrations were available at the 48–72 h post-injury sampling timepoint. ROC curve analysis for these variables in this patient cohort generated AUC values of 0.763 (95%CI, 0.639–0.887) for resistin and 0.707 (95%CI, 0.573–0.841) for CRP (Figure 2B).

### 3.4. Post-Injury Elevations in Plasma Resistin Levels Are Positively Associated with Markers of Systemic Inflammation

A recent study of 38 adult burns patients reported that resistin forms a network with inflammatory cytokines during the acute post-injury phase [29]. In line with this, we found plasma resistin concentrations positively correlated with the circulating levels of G-CSF, IL-6, and IL-8 in burns patients on day 1 of injury (Table 2). In the case of G-CSF and IL-6, this relationship persisted at day 3 post-burn, where circulating concentrations of TNF-α were also positively correlated with resistin levels (Table 2). At day 28 post-burn, positive relationships were also detected between plasma concentrations of resistin, G-CSF, and IL-8 (Table 2).

In our cohort of major trauma patients, plasma resistin levels positively correlated with concentrations of IL-6, IL-8, IL-10, G-CSF, TNF-α, and MCP-1 at all three post-injury sampling time-points (Table 3).

### 3.5. PBMCs and Neutrophils Secrete Resistin Following LPS Stimulation

**Figure 3:**
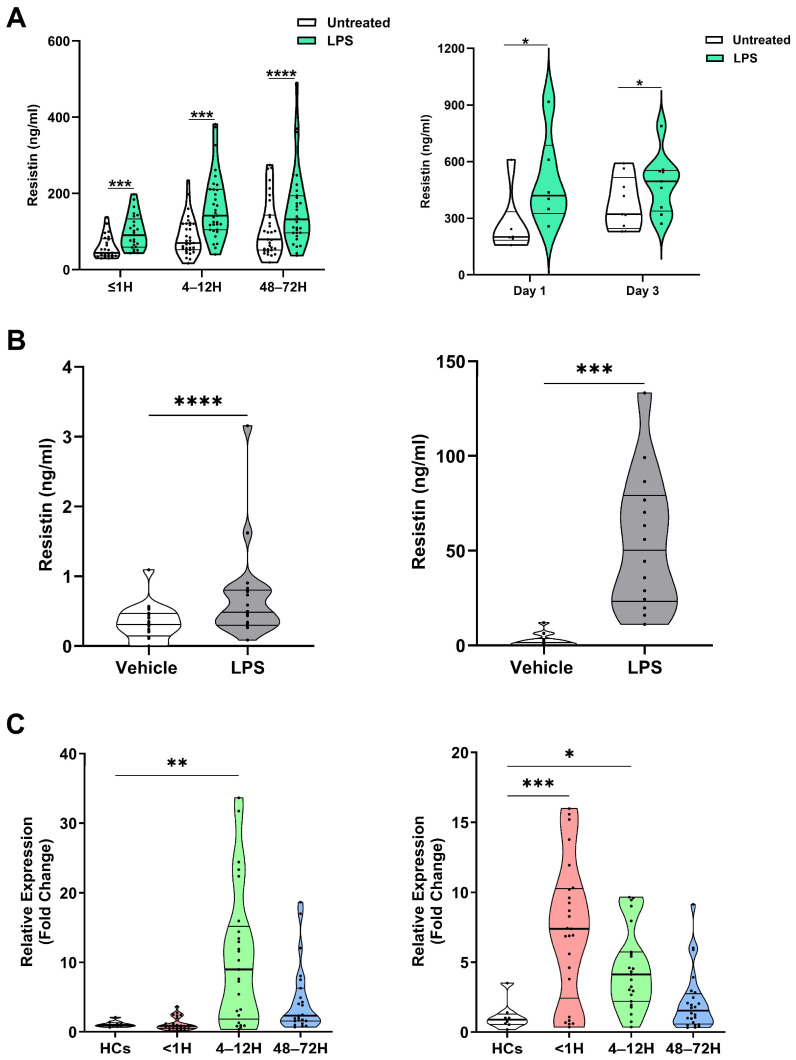
Leukocytes secrete resistin upon lipopolysaccharide (LPS) stimulation. (**A**) **Left** panel: Concentrations of resistin measured in supernatants of whole blood samples acquired from trauma patients at three post-injury timepoints (≤1, 4–12, and 48–72 h) following a 4 h stimulation with 10 ng/mL LPS or vehicle control (≤1 h, *n* = 25; 4–12 h, *n* = 33; and 48–72 h, *n* = 31). *** *p* < 0.0005, **** *p* < 0.0001. **Right** panel: Concentrations of resistin measured in supernatants of whole blood samples acquired from thermally-injured patients at day 1 (*n* = 6) and day 3 (*n* = 9) post-burn following a 4 h stimulation with 10 ng/mL LPS or vehicle control. * *p* < 0.05. (**B**) **Left** panel; concentrations of resistin measured in supernatants collected from cultures of isolated PBMCs from healthy controls (HCs; *n* = 20) following a 4 h stimulation with 10 ng/mL LPS or vehicle control. **Right** panel, concentrations of resistin measured in supernatants collected from cultures of isolated neutrophils from HCs (*n* = 14) following a 2 h stimulation with 100 ng/mL LPS or vehicle control. *** *p* < 0.0005, **** *p* < 0.0001. (**C**) **Left** panel; comparison of resistin gene expression in resting peripheral blood mononuclear cells (PBMCs) isolated from HCs (*n* = 9) and major trauma patients at three post-injury timepoints (≤1 h, *n* = 25; 4–12 h, *n* = 25; and 48–72 h, *n* = 23). ** *p* < 0.005 vs. HCs. **Right** panel; comparison of resistin gene expression in resting neutrophils isolated from HCs (*n* = 8) and major trauma patients at three post-injury timepoints (≤1 h, *n* = 25; 4–12 h, *n* = 23; and 48–72 h, *n* = 24). * *p* < 0.05, ***p < 0.0005 vs. HCs.

To investigate potential origins of circulating resistin post-injury, we measured resistin concentrations in supernatants collected from whole blood samples of trauma and burns patients following a 4 h stimulation with LPS. Revealing leukocytes to be a source of resistin, we found resistin concentrations were significantly higher in supernatants from LPS-challenged blood samples when compared to vehicle treated controls (Figure 3A). Supporting this observation that resistin production by PBMCs and neutrophils may contribute to systemic hyperresistinemia following major injury, we found that LPS challenge in vitro also promoted resistin production by purified PBMCs and neutrophils isolated from HCs (Figure 3B).

Analysis of resistin gene expression in PBMCs and neutrophils isolated from traumatically-injured patients and HCs revealed significantly higher resistin mRNA levels in PBMCs at our 4–12 h post-injury sampling timepoint and in neutrophils isolated from patients ≤1 and 4–12 h post-trauma (Figure 3C).

### 3.6. Inflammatory Agonists, Including mtDAMPs, Promote Resistin Secretion by Unprimed and Primed Neutrophils

**Figure 4:**
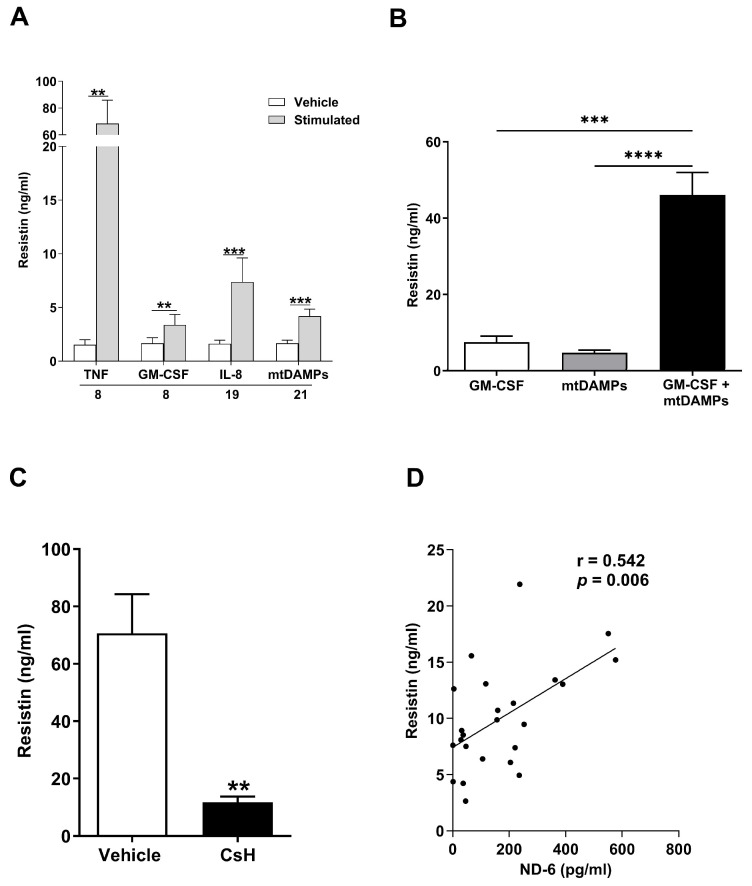
Mitochondrial-derived damage-associated molecular patterns (MtDAMPs) promote secretion of resistin from neutrophils in a formyl peptide receptor-1 (FPR-1) dependent manner. (**A**) Concentrations of resistin measured in supernatants collected from unprimed neutrophils isolated from healthy controls following a 2 h stimulation with 10 ng/mL tumour necrosis factor-alpha (TNF-α; *n* = 8), 10 ng/mL granulocyte macrophage colony stimulating factor (GM-CSF, *n* = 8), 100 ng/mL Interleukin (IL)-8 (*n* = 19) or 100 µg/mL mtDAMPs (*n* = 21). ** *p* < 0.005, *** *p* < 0.0005. (**B**) Concentrations of resistin measured in supernatants obtained from neutrophils subjected to the following treatments; GM-CSF (10 ng/mL) for 60 min, MtDAMPs (100 µg/mL) for 60 min or GM-CSF (10 ng/mL) for 30 min followed by MtDAMPs (100 µg/mL) for 30 min (*n* = 11). *** *p* < 0.0005, **** *p* < 0.0001. (**C**) Concentrations of resistin measured in supernatants collected from cultures of GM-CSF primed neutrophils treated for 1 h with 1 µM of the FPR-1 specific inhibitor cyclosporin H (CsH) or vehicle control prior to a 30 min stimulation with 100 µg/mL MtDAMPs (*n* = 8). ** *p* < 0.005. (**D**) Correlative analysis examining the relationship between the circulating concentrations of resistin and the N-formylated peptide ND6 in blood samples obtained from major trauma patients (*n* = 24) within 1 h of injury.

Major traumatic and thermal injuries are a sterile insult that promote systemic inflammation and result in significant cellular and tissue damage. As such, we investigated how exposure to a variety of inflammatory agonists influenced resistin secretion by neutrophils, the most abundant leukocyte in human circulation. Relative to vehicle controls, we detected significantly higher resistin concentrations in culture supernatants derived from unprimed neutrophils treated with TNF-α, GM-CSF, IL-8, or mtDAMPs (Figure 4A).

To mimic the post-injury inflammatory environment more closely, we subsequently performed priming experiments where neutrophils were treated with GM-CSF prior to stimulation with mtDAMPs. As shown in Figure 4B, GM-CSF and mtDAMP co-treated neutrophils secreted greater amounts of resistin when compared to neutrophils challenged with either stimulus alone.

Pointing towards N-formylated peptides as the prominent agonist stimulating resistin secretion by mtDAMP-treated neutrophils, we found that prior exposure of neutrophils to the FPR-1 inhibitor CsH significantly reduced the amount of resistin released by GM-CSF primed neutrophils challenged with whole mtDAMP preparations (Figure 4C). Previously, we have reported that circulating concentrations of the N-formylated peptide ND6 are significantly elevated within minutes of injury [23]. Strengthening the idea that the release of N-formylated peptides from damaged tissue may promote resistin secretion by neutrophils post-injury, we found plasma concentrations of ND6 in pre-hospital blood samples were positively associated with circulating levels of resistin (Figure 4D).

### 3.7. Elevated Plasma Concentrations of Resistin Post-Trauma Are Associated with the Induction of Endotoxin Tolerance and Impaired Neutrophil Anti-Microbial Responses

**Figure 5:**
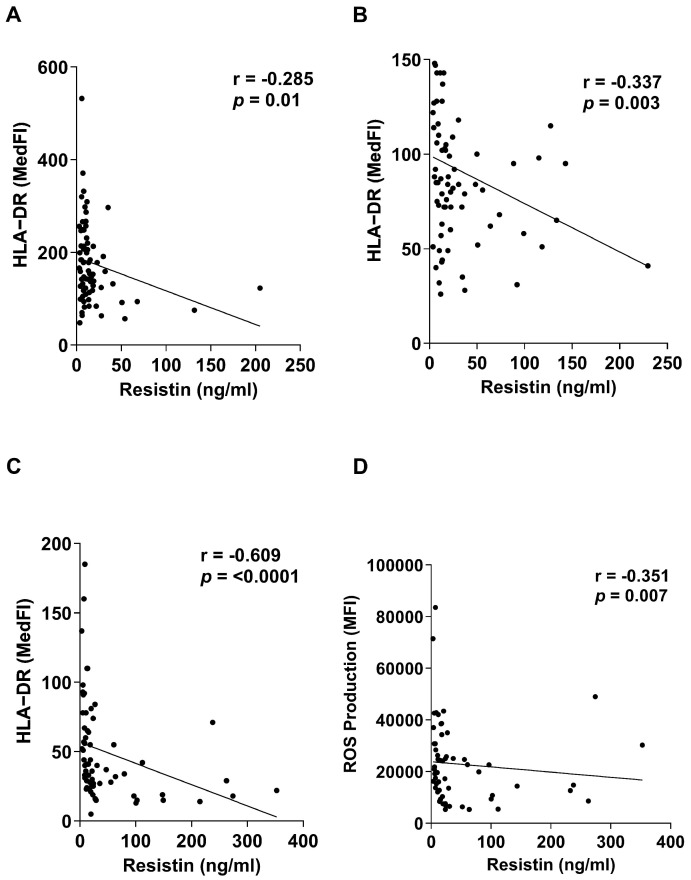
Elevated concentrations of plasma resistin are associated with markers of immune modulation following major traumatic injury. (**A**–**C**) Correlative analysis examining the relationship between plasma concentrations of resistin and expression of HLA-DR on the surface of monocytes isolated from trauma patients at three post-injury timepoints (≤1 h, *n* = 80; 4–12 h, *n* = 75; 48–72 h, *n* = 66). (**D**) Correlative analysis showing the negative association between plasma concentrations of resistin and ex vivo phorbol myristate acetate (PMA)-induced reactive oxygen species (ROS) production by neutrophils isolated from major trauma patients 48–72 h post-injury (*n* = 58).

When compared to HCs, we have previously demonstrated that whole blood leukocytes isolated from trauma patients secrete significantly less TNF-α and IL-6 following LPS stimulation, with this functional impairment accompanied by reduced expression of HLA-DR on the surface of monocytes [2]. When correlating these data with paired measurements of plasma resistin levels, we found that TNF-α and IL-6 production by LPS-challenged leukocytes was negatively associated with resistin concentrations across our three study timepoints (Table 4). Moreover, we observed a negative relationship between plasma resistin levels and the density of the antigen-presenting molecule HLA-DR on the surface of monocytes, (Figure 5A–C). No associations were observed between plasma resistin concentrations and production of the anti-inflammatory cytokine IL-10 by LPS-challenged whole blood leukocytes (Table 4).

Alongside the induction of endotoxin tolerance, we have shown that neutrophils exhibit impaired anti-microbial responses post-trauma, with PMA-induced ROS production significantly reduced when compared to HCs, with the greatest degree of impairment observed 48–72 h post-injury [2]. As shown in Figure 5D, we found plasma resistin concentrations 48–72 h post-injury were negatively associated with the ability of neutrophils to generate ROS in response to PMA stimulation.

### 3.8. Resistin Treatment Significantly Reduces LPS-Induced Cytokine Production by THP-1 Cells and PMA-Induced ROS Production by Neutrophils Isolated from HCs

**Figure 6:**
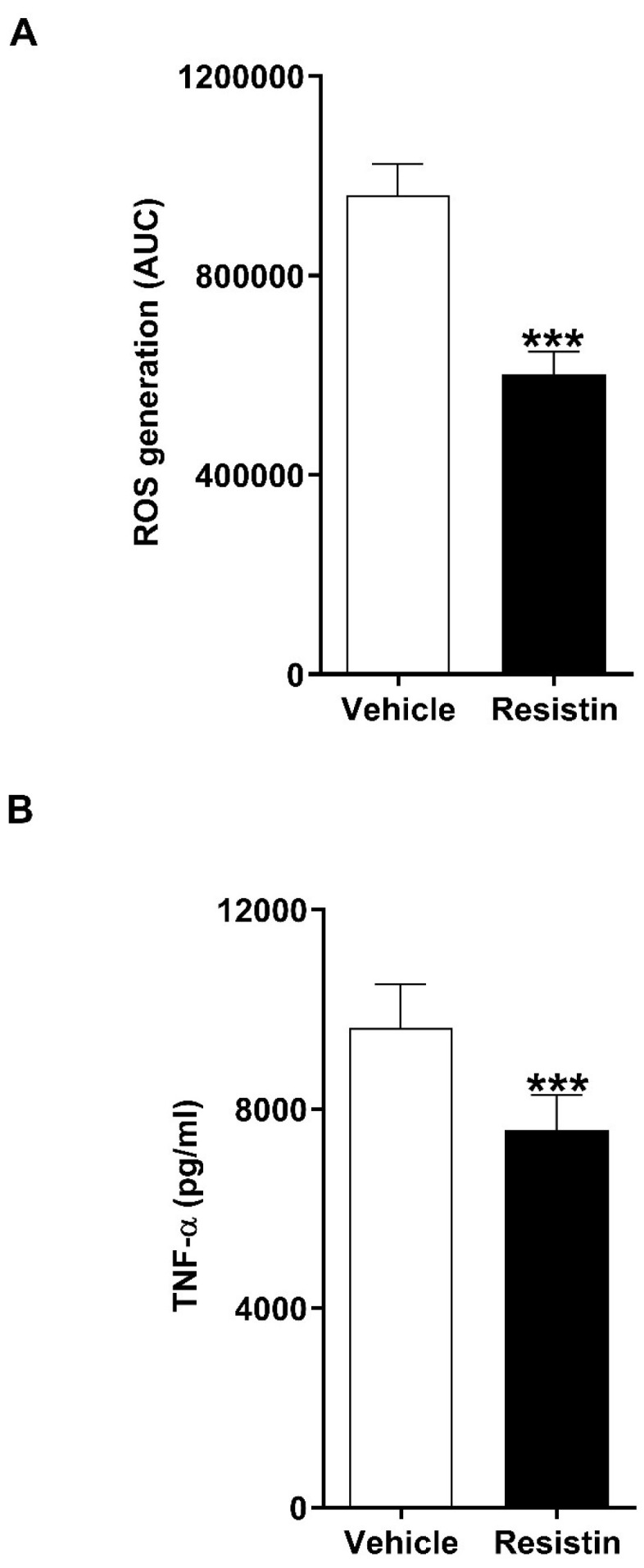
In vitro treatment with resistin significantly impairs the anti-microbial functions of neutrophils and THP-1 cells. (**A**) Phorbol myristate acetate-induced reactive oxygen species (ROS) production by neutrophils isolated from healthy controls following a 1 h pre-treatment with 150 ng/mL resistin or vehicle control (*n* = 9). (**B**) Lipopolysaccharide-induced tumour necrosis factor-alpha (TNF-α) production by THP-1 cells pre-treated for 24 h with 150 ng/mL resistin or vehicle control (*n* = 9). *** *p* < 0.0005.

Previous studies have demonstrated that in vitro exposure to supraphysiological doses of resistin inhibits the anti-microbial responses of neutrophils and monocytes [17,18,19,21]. To investigate whether resistin exhibits immune suppressive properties at concentrations detectable in patients, we treated neutrophils and THP-1 cells with the peak concentration of resistin we measured in thermally-injured patients (150 ng/mL) before assessing their ability to generate ROS in response to PMA stimulation and secrete TNF-α upon LPS challenge, respectively. As shown in Figure 6A, pre-treatment of neutrophils with resistin significantly reduced their generation of ROS following PMA activation. Similarly, compared to vehicle-treated controls, prior exposure of THP-1 cells to resistin significantly impaired their production of TNF-α when challenged with LPS (Figure 6B).

## 4. Discussion

Predisposing patients to an increased risk of mortality and the development of such secondary complications as sepsis, acute respiratory distress syndrome, and multiple organ failure, a state of immediate and prolonged systemic immune suppression is a well- described consequence of major traumatic and thermal injury [4,10,11,12,40,41,42]. However, despite its clinical impact, the mechanisms that promote and sustain a dysregulated CARS response in these patient groups is poorly understood. In the current study, we analysed serial blood samples acquired from over 200 major trauma and burns patients across the ultra-early (≤1 h), acute (4–72 h), and long-term (days 14–28) injury phases. We report an immediate and persistent elevation in circulating concentrations of resistin, which may contribute to the impaired neutrophil and monocyte anti-microbial responses that characterise the injury-induced dysregulated CARS response that renders hospitalised trauma patients susceptible to poor clinical outcomes.

To date, studies that have reported a state of systemic hyperresistinemia in severely injured adults have analysed blood samples obtained from patients across a timeframe that spans from the point of hospital admission to day 14 post-injury [25,27,28,29,30,31]. Consequently, no data existed on circulating resistin levels in the pre-hospital or long-term injury setting, with studies highlighting the need to address this knowledge gap in order to establish whether resistin is a potential therapeutic target by which to prevent and/or treat the prolonged pathophysiological responses, immune suppression, and chronic critical illness experienced by survivors of major injury [29,34,43]. Here, we have shown, for the first time, that hyperresistinemia is detectable during the immediate injury-induced inflammatory response, with resistin concentrations significantly higher in plasma samples acquired from major trauma patients within 1 h of injury when compared to HCs. Furthermore, in addition to confirming that burns patients present with significantly elevated levels of circulating resistin at the time of hospital admission to day 14 post-injury [25,27,29], we have demonstrated that this state of systemic hyperresistinemia persists for up to 28 days post-burn.

Recent retrospective and prospective cohort studies in major adult and paediatric burns patients have reported that plasma resistin levels positively correlate with a range of pro-inflammatory cytokines and acute phase proteins (e.g., IL-6, IL-8, MCP-1, C-reactive protein) [26,29]. In agreement, we found, in both our trauma and burns cohorts, that plasma resistin concentrations positively associated with the circulating levels of G-CSF, IL-6, IL-8, MCP-1, and TNF-α across the ultra-early, acute and/or sub-acute injury phases. Whilst not able to demonstrate causality directly, these data suggest that a positive feedback loop may exist between the production of pro-inflammatory cytokines and resistin in critically ill patients. In support of this suggestion, in vitro studies have demonstrated that, through activation of nuclear factor kappa-B (NFκB), resistin stimulation of leukocytes increases their production of IL-6, IL-8, and TNF-α [15,18,44], whereas exposure to cytokines, such as TNF-α, increased resistin expression in monocytes, macrophages, neutrophils, and PBMCs [15,45,46,47,48,49]. In line with these latter studies, we showed here that treating neutrophils with TNF-α, GM-CSF, or IL-8 significantly increased their production of resistin.

Confirming a recent observation in a smaller study of adult burns patients [29], we found no association between circulating resistin concentrations and BMI scores in our thermally-injured cohort, implying that adipose tissue is not a source of circulating resistin post-burn. Indeed, resistin gene expression was previously reported to be comparable in adipose tissue biopsies obtained from severe burns patients and uninjured controls [27]. Suggesting that leukocytes are a potential source of plasma resistin, we found that LPS challenge of whole blood samples obtained from both trauma patients and HCs resulted in the secretion of resistin, whilst resistin mRNA levels were significantly higher in purified neutrophil and PBMC populations isolated from patients ≤1 and/or 4–12 h post-injury. This latter observation aligns with the findings of Duffy and colleagues who reported resistin gene expression was significantly increased in peripheral blood monocytes acquired from burns patients 12–24 h post-injury [27]. Macrophages are also known to express resistin [47], suggesting that they could contribute to the state of hyperresistinemia that develops post-injury. As we did not collect tissue biopsy samples from our traumatically-injured patients, we were unable to investigate this possibility in our study.

In vitro exposure to TNF-α, LPS, or N-Formylmethionyl-leucyl-phenylalanine (fMLP) has been shown to increase resistin production by neutrophils isolated from HCs [13,14,45]. Adding to this list of agonists that can trigger resistin secretion by neutrophils, we found that when compared to vehicle treated controls, resistin concentrations were significantly higher in supernatants collected from cultures of GM-CSF, IL-8, or mtDAMP- challenged neutrophils, with primed neutrophils secreting significantly more resistin than their unprimed counterparts. Pointing towards N-formylated peptides as the prominent agonist stimulating resistin secretion by mtDAMP-treated neutrophils, we found that prior treatment with the FPR-1 inhibitor CsH significantly reduced the amount of resistin released by GM-CSF primed neutrophils challenged with whole mtDAMP preparations. Adding strength to this argument was our observation of a positive association between plasma levels of resistin and the concentration of the mitochondrial-derived N-formylated peptide ND6 in blood samples acquired from major trauma patients ≤1 h post-injury. Interestingly, previous work has also shown that exposure to the nuclear-derived DAMP, high mobility group box-1 (HMGB-1), can induce resistin production by monocytes [50]. Thus, taken together, these results suggest that immune activation, secondary to tissue damage and the release of DAMPs, may be a potential mechanism for how a state of systemic hyperresistinemia develops following major injury.

Resistin is an immunomodulatory molecule that possesses both immune activatory and suppressive properties [17,18,19,20,21,22,51,52]. Focussing upon its immune suppressive actions, in vitro studies have shown that prior exposure to resistin induces endotoxin tolerance in monocytes [17,18] and decreases neutrophil ROS production and chemotactic activity [19,21,22]. As these impairments in immune function mirror the behaviour of innate immune cells isolated from trauma and burns patients [2,4,7,8,24,53], *could elevated concentrations of resistin contribute to injury-induced immune dysfunction?* Although our data does not establish resistin as a direct causative agent of injury-induced immune modulation, we did observe, in our cohort of major trauma patients, negative relationships between plasma resistin levels and (i) TNF-α and IL-6 production by LPS-challenged leukocytes, (ii) the density of HLA-DR molecules on the surface of monocytes, and (iii) PMA-induced ROS generation, three features of the CARS response triggered by severe injury [7,8,9,24,54]. As we found that exposure to LPS triggers resistin secretion by whole blood leukocytes and purified PBMCs, it is conceivable that a feedback loop could develop in times of infectious challenge in hospitalised trauma patients. As a ligand of TLR4 [17], it could be that resistin secreted by LPS-stimulated leukocytes competes with LPS for occupancy of this pathogen recognition receptor [18]. As such, this could reduce monocyte responses to LPS challenge and thereby offer a potential mechanism by which to explain how endotoxin tolerance develops in severely-injured patients.

Given that the post-injury sampling of our trauma patient cohort was restricted to the 1–72 h post-injury window, we do not know whether the negative associations found between resistin concentrations and innate immune cell function persisted in the weeks following trauma where a state of systemic hyperresistinemia and immune modulation remains. Addressing this knowledge gap should be the focus of future research given that such data would potentially help us understand whether targeting resistin could aid in the prevention and/or treatment of the debilitating long-term sequelae experienced by survivors of major injury, which includes chronic immune suppression and recurrent hospital-acquired infections [34]. Related to this, it would also be of interest for future studies to examine whether a state of systemic hyperresistinemia contributes to the functional impairments that have been described in the adaptive arm of the immune system following severe trauma [55,56,57,58].

Studies that have assigned immune suppressive properties to resistin have often used supraphysiological concentrations in their experimental assays (100–1000 ng/mL), with the doses chosen exceeding the levels recorded in the circulation of both HCs and critically ill patients (1–35 ng/mL) [17,18,19,21,25,26,27,28]. Thus, to provide physiological relevance to our study, we investigated the impact that in vitro exposure to concentrations of resistin measured in plasma samples of burns patients had on the anti-microbial activities of neutrophils and monocytes. Compared to vehicle controls, we found resistin-treated neutrophils and THP-1 cells exhibited impaired PMA-induced ROS generation and LPS-induced cytokine production respectively. Potential mechanisms that may explain these impaired responses include resistin-mediated interference of LPS binding to its receptor, toll-like receptor 4, on the monocyte surface [17,18], reduced activation of PI3K [19], and impaired actin polymerisation [20]. Whilst demonstrating the immunomodulatory properties of resistin on innate immune cell function, future studies should treat primary PBMCs isolated from HCs with resistin to examine its tolerising effects on LPS-induced cytokine production in order to overcome the inherent limitations that are associated with using the THP-1 cell line when attempting to study human monocyte function.

Although suppressive, the immune inhibitory effects we observed for resistin were relatively small. As such, our results suggest resistin is a potential factor that contributes to the state of systemic immune modulation post-trauma and is not the sole driving factor. Indeed, we and others have measured, during the ultra-early and acute post-injury phases, elevated levels of anti-inflammatory cytokines as well as immune suppressive and tolerising DAMPs, such as heme, HMGB-1, prostaglandin E_2_, and mitochondrial-derived DNA [1,2,3,6,59,60,61]. Moreover, endogenous production of catecholamines during the injury-induced stress response, as well as injury-specific factors such as severity, shock, and ischemia/reperfusion may also contribute to injury-induced immune dysregulation. Thus, in conjunction with resistin, these factors would create an immunomodulatory milieu that promotes modulation of the innate immune response in the minutes, hours, and days following injury.

A recent meta-analysis of ten studies that examined resistin levels in critically ill patients found a significant increase in resistin levels amongst intensive care unit patients when compared to healthy controls and suggested that hyperresistinemia may contribute to organ dysfunction and poor clinical outcomes [62]. In agreement with this, when we compared resistin levels between trauma patients who did or did not develop MODS, we found that those who experienced this secondary complication presented with significantly higher plasma concentrations of resistin 4–12 and 48–72 h post-injury. In traumatically brain-injured patients, a state of hyperresistinemia has been reported in non-survivors and those who experienced poor long-term functional recovery [28,31,32], leading to the suggestion that resistin could potentially serve as a useful predictor of poor clinical outcomes [28,32]. Adding support to this, we found, in our trauma patient cohort, that ROC curves built on plasma resistin levels measured 48–72 h post-injury had good discriminatory power for distinguishing between patients who did or did not develop MODS. However, it must be noted that this result was derived from data collected from a patient cohort recruited at a single major trauma centre, meaning our findings require validation in independent prospective-based studies to determine both its validity and generalisability.

## 5. Conclusions

We report that major traumatic and severe thermal injuries result in an immediate (≤1 h) and persistent (28 days) elevation in plasma concentrations of resistin, and that systemic hyperresistinemia is associated with the development of MODS post-trauma. Furthermore, we have provided evidence that suggests (i) leukocytes are a source of circulating resistin, (ii) exposure to mtDAMPs can promote resistin secretion by neutrophils and (iii) elevated levels of resistin may contribute to the innate immune dysregulation that develops in the hours, days, and weeks following major injury. Our work has therefore begun to address the call for a better understanding of resistin in the critical care setting, which is needed if we are to establish whether it represents a potential biomarker of poor outcomes and a target by which to prevent and/or treat the chronic pathophysiology experienced by survivors of severe trauma.

## Figures and Tables

**Table 1:** Trauma and burns patient demographics. Data are expressed as mean (range) unless otherwise stated. ^#^ Information relating to ISS for patients enrolled into the BBATS study was available for 137 patients. ^@^ Due to the definition adopted, a diagnosis of MODS was available for a total of 121 patients enrolled into the BBATS study. ABSI, Abbreviated burn severity index; A/P, Assault/Penetrating; GCS, Glasgow coma scale; ICU, Intensive care unit; ISS, Injury severity score; MODS, Multiple organ dysfunction syndrome; RTC, Road traffic collision; TBSA, Total body surface area; TBSA FT, Total body surface area full thickness.

Characteristic	Trauma Patients (*n* = 147)	Burns Patients (*n* = 95)
Age, years (range)	42 (18–95)	47 (16–83)
Sex, (M:F)	126:21	75:20
Time to pre-hospital sample, minutes post-injury (range)	42 (13–60)	-
ISS (range) ^#^	25 (9–66)	-
Admission GCS score (range)	10 (3–15)	-
Mechanism of injury Fall, *n* (%) A/P, *n* (%) Blunt, *n* (%) RTC, *n* (%) Flash, *n* (%) Flame, *n* (%) Flame and flash, *n* (%) Electrical, *n* (%) Scald, *n* (%)	30 (20) 32 (22) 7 (5) 78 (53) - - - - -	- - - - 7 (8) 79 (83) 5 (5) 1 (1) 3 (3)
% TBSA (range)	-	36 (15–85)
% TBSA FT (range)	-	18.6 (0–80)
ABSI (range)	-	8 (2–14)
Baux (range)	-	82 (34–143)
rBaux (range)	-	90 (39–160)
MODS, *n* (%)	63 (52) ^@^	-
ICU-free days (range)	19 (0–30)	16 (0–30)
Hospital-free days (range)	7 (0–29)	4 (0–24)
Mortality, *n* (%)	24 (16.3)	19 (20)

**Table 2:** Correlative analyses investigating the relationships between plasma resistin levels and the circulating concentrations of pro- and anti-inflammatory cytokines in severe burns patients at five post-burn sampling timepoints. Significant associations are indicated in bold font. *p* values presented are adjusted for false discovery rate using Benjamini–Hochberg corrections. G-CSF, Granulocyte colony-stimulating factor; IL, Interleukin; MCP-1, Monocyte chemoattractant protein-1; TNF-α, Tumour necrosis factor-alpha.

	Day 1	Day 3	Day 7	Day 14	Day 28
G-CSF	**R = 0.489**	**R = 0.459**	R = 0.002	R = −0.069	**R = 0.423**
	***p*** ** = 0.0003**	***p*** ** = 0.002**	*p* = 0.987	*p* = 0.820	***p*** ** = 0.040**
	***n*** ** = 56**	***n*** ** = 48**	*n* = 48	*n* = 41	***n*** ** = 32**
IL-6	**R = 0.459**	**R = 0.339**	R = 0.034	R = −0.003	R = 0.389
	***p*** ** = 0.0007**	***p*** ** = 0.032**	*p* = 0.954	*p* = 0.984	*p* = 0.064
	***n*** ** = 56**	***n*** ** = 48**	*n* = 48	*n* = 41	*n* = 32
IL-8	**R = 0.497**	R = 0.171	R = 0.056	R = 0.019	**R = 0.540**
	***p*** ** = 0.0002**	*p* = 0.270	*p* = 0.954	*p* = 0.984	***p*** ** = 0.005**
	***n*** ** = 56**	*n* = 48	*n* = 48	*n* = 41	***n*** ** = 32**
IL-10	R = −0.06	R = 0.254	R = 0.039	R = 0.057	R = 0.080
	*p* = 0.741	*p* = 0.112	*p* = 0.954	*p* = 0.839	*p* = 0.694
	*n* = 56	*n* = 48	*n* = 48	*n* = 41	*n* = 32
MCP-1	R = 0.044	R = 0.244	R = 0.060	R = 0.086	R = 0.038
	*p* = 0.823	*p* = 0.115	*p* = 0.954	*p* = 0.776	*p* = 0.833
	*n* = 56	*n* = 48	*n* = 48	*n* = 41	*n* = 32
TNF-a	R = 0.016	**R = 0.364**	R = 0.074	R = 0.012	R = 0.259
	*p* = 0.915	***p*** ** = 0.020**	*p* = 0.954	*p* = 0.985	*p* = 0.198
	*n* = 56	***n*** ** = 48**	*n* = 48	*n* = 41	*n* = 32

**Table 3:** Correlative analyses investigating the relationships between plasma resistin levels and the circulating concentrations of pro- and anti-inflammatory cytokines in major trauma patients at three post-injury sampling timepoints. Significant associations are indicated in bold font. *p* values presented are adjusted for false discovery rate using Benjamini–Hochberg corrections. G-CSF, Granulocyte colony-stimulating factor; IL, Interleukin; MCP-1, Monocyte chemoattractant protein-1; TNF-α, Tumour necrosis factor-alpha.

	≤1 h	4–12 h	48–72 h
G-CSF	**R = 0.249**	**R = 0.722**	**R = 0.356**
	***p*** ** = 0.018**	***p*** ** = <0.0001**	***p*** ** = 0.003**
	***n*** ** = 89**	***n*** ** = 81**	***n*** ** = 72**
IL-6	**R = 0.652**	**R = 0.693**	**R = 0.342**
	***p*** ** = <0.0001**	***p*** ** = <0.0001**	***p*** ** = 0.004**
	***n*** ** = 89**	***n*** ** = 81**	***n*** ** = 72**
IL-8	**R = 0.559**	**R = 0.786**	**R = 0.464**
	***p*** ** = <0.0001**	***p*** ** = 0**	***p*** ** = <0.0001**
	***n*** ** = 89**	***n*** ** = 81**	***n*** ** = 72**
IL-10	**R = 0.694**	**R = 0.284**	**R = 0.464**
	***p*** ** = <0.0001**	***p*** ** = 0.014**	***p*** ** = <0.0001**
	***n*** ** = 89**	***n*** ** = 81**	***n*** ** = 72**
MCP-1	**R = 0.674**	**R = 0.594**	**R = 0.517**
	***p*** ** = <0.0001**	***p*** ** = <0.0001**	***p*** ** = <0.0001**
	***n*** ** = 89**	***n*** ** = 81**	***n*** ** = 72**
TNF-α	**R = 0.574**	R = 0.153	**R = 0.465**
	***p*** ** = <0.0001**	*p* = 0.241	***p*** ** = <0.0001**
	***n*** ** = 89**	*n* = 81	***n*** ** = 72**

**Table 4:** Correlative analyses examining the relationship between plasma resistin levels and the concentrations of tumour necrosis factor-alpha (TNF-α), interleukin (IL)-6 (IL-6), and IL-10 produced by ex vivo lipopolysaccharide-challenged whole blood samples acquired from major trauma patients at three post-injury sampling timepoints. Confidence intervals of 95% are presented in parentheses. Significant associations according to Spearman’s rank correlation coefficient are indicated in bold font. IL, Interleukin; TNF-α, Tumour necrosis factor-alpha.

	≤1 h	4–12 h	48–72 h
IL-6	**R = −0.283**	**R = −0.494**	**R = −0.609**
	**(−0.526–0.004)**	**(−0.689–−0.233)**	**(−0.781–−0.350)**
	***p*** ** = 0.047**	***p*** ** = 0.0004**	***p*** ** = <0.0001**
	***n*** ** = 50**	***n*** ** = 47**	***n*** ** = 38**
TNF-α	R = −0.261	**R = −0.477**	**R = −0.548**
	(−0.509–0.03)	**(−0.677–−0.212)**	**(−0.743–−0.268)**
	*p* = 0.067	***p*** ** = 0.0007**	***p*** ** = 0.0004**
	*n* = 50	***n*** ** = 47**	***n*** ** = 38**
IL-10	R = 0.096	R = −0.113	R = −0.021
	(−0.196–0.372)	(−0.394–0.188)	(−1–1)
	*p* = 0.508	*p* = 0.450	*p* = 0.898
	*n* = 50	*n* = 47	*n* = 38

## Data Availability

The original contributions presented in this study are included in the article/Supplementary Materials. Further inquiries can be directed to the corresponding author(s).

## Supplementary Materials

The following supporting information can be downloaded at: https://www.mdpi.com/article/10.3390/biom16030443/s1, Figure S1: Impact of gender on the circulating concentrations of resistin in traumatic and thermally-revisedinjured patients; Table S1: Correlative analyses examining the relationship between circulating resistin levels, age and body mass index (BMI) in major trauma and thermally-injured patients; Table S2: Characteristics of trauma patients that did or not develop post-injury multiple organ dysfunction syndrome (MODS).

## Abbreviations

ABSI	Abbreviated Burn Severity Index
AUC	Area under the curve
AUROC	Area under the receiver operating characteristic curve
BBATS	Brain Biomarkers after Trauma Study
BMI	Body Mass Index
CARS	Compensatory Anti-inflammatory Response Syndrome
CM	Complete Media
CsH	Cyclosporin H
FDR	False Discovery Rate
FIZZ	Found in Inflammatory Zones
fMLP	N-Formylmethionyl-leucyl-phenylalanine
FPR-1	Formyl Peptide Receptor-1
GCS	Glasgow Coma Scale
G-CSF	Granulocyte Colony-Stimulating Factor
GM-CSF	Granulocyte-Macrophage Colony-Stimulating Factor
GPS	Glutamine Penicillin Streptomycin
HBSS	Hanks Balanced Salt Solution
HCs	Healthy Controls
HLA-DR	Human Leukocyte Antigen-DR
HMGB-1	High Mobility Group Box-1
ICU	Intensive Care Unit
IL	Interleukin
ISS	Injury Severity Score
LPS	Lipopolysaccharide
MCP	Monocyte Chemoattractant Protein
MedFI	Median Fluorescence Intensity
MFI	Mean Fluorescence Intensity
MODS	Multiple Organ Dysfunction Syndrome
mRNA	Messenger RNA
MtDAMPs	Mitochondrial-derived damage-associated molecular patterns
ND6	NADH dehydrogenase 6
NFκβ	Nuclear Factor Kappa Beta
PBS	Phosphate buffered saline
PFP	Platelet-free plasma
PMA	Phorbol 12-myristate-13-acetate
REC	Research Ethics Committee
RPMI	Roswell Park Memorial Institute Media
ROS	Reactive Oxygen Species
RT	Room Temperature
RT-PCR	Real Time-Polymerase Chain Reaction
SIFTI	Scientific Investigation Following Thermal Injury
SIRS	Systemic Inflammatory Response Syndrome
SOFA	Sequential Organ Failure Assessment
TBI	Traumatic Brain Injury
TBSA	Total Body Surface Area
TNF	Tumour Necrosis Factor
UHBFT	University Hospitals Birmingham Foundation Trust
WMRBC	West Midlands Regional Burns Centre
