# Supplementary material for: Major Traumatic and Severe Thermal Injuries Lead to Immediate and Persistent Elevations in Circulating Concentrations of Resistin That Are Associated with Poor Clinical Outcomes and Impaired Innate Immune Responses
Source: Biomolecules. 2026 Mar 16;16(3):443. doi: 10.3390/biom16030443 (PMC13023763; doi:10.3390/biom16030443)
Supplement: Supplementary file 1 [file biomolecules-16-00443-s001.zip › biomolecules-4144212-supplementary.pdf]

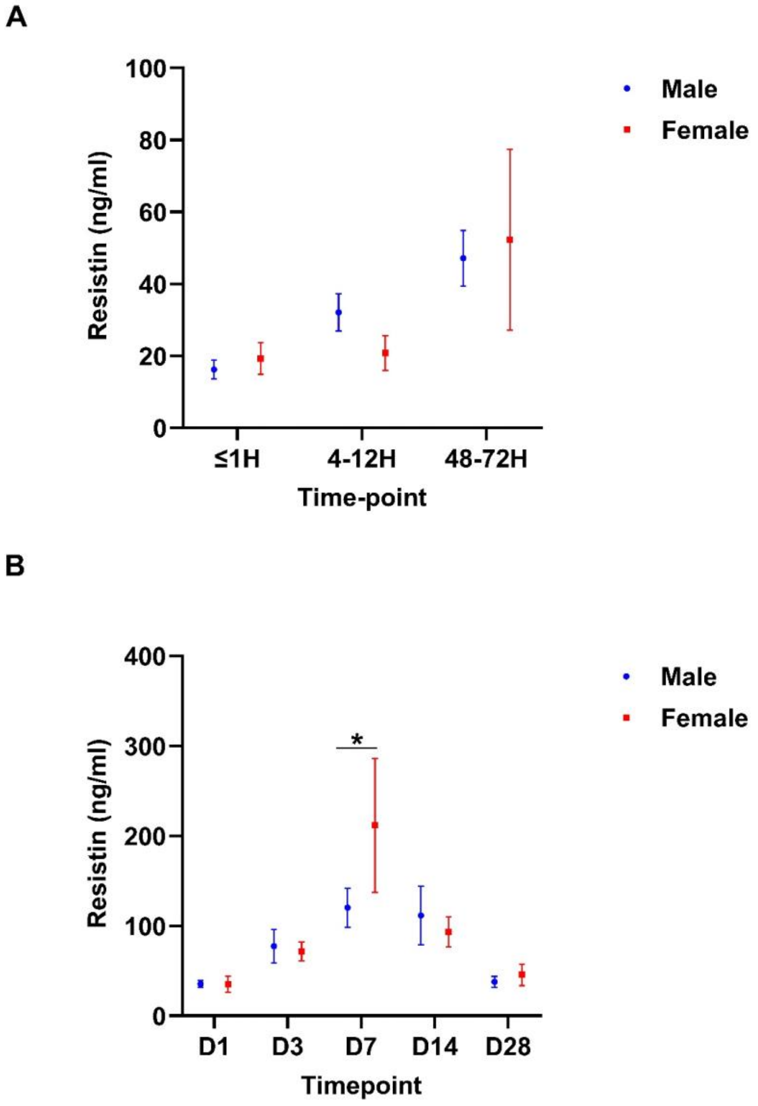

Figure S1. Impact of gender on the circulating concentrations of resistin in traumatic and thermally-injured patients. **(A)** Comparison of plasma resistin concentrations between male and female patients ≤1 h (Male, $n = 102$; female, $n = 18$), 4–12 hours (Male, $n = 102$; female, $n = 16$) and 48–72 hours (Male, $n = 93$; female, $n = 14$) after sustaining a major traumatic injury. **(B)** Comparison of plasma resistin concentrations between male and female patients on days 1 (Male, $n = 73$; female, $n = 20$), 3 (Male, $n = 67$; female, $n = 17$), 7 (Male, $n = 56$; female, $n = 18$), 14 (Male, $n = 50$; female, $n = 16$) and 28 (Male, $n = 39$; female, $n = 15$) after sustaining a severe thermal injury. *$p < 0.05$.

Table S1. Correlative analyses examining the relationship between circulating resistin levels, age and body mass index (BMI) in major trauma and thermally-injured patients. *P* values presented are adjusted for false discovery rate using Benjamini-Hochberg corrections. Significant associations are indicated in bold font. BMI, Body Mass Index.

	**Trauma patients**				
	**≤1 h**	**4–12 h**	**48–72 h**		
**Age**	R = −0.132<br>*p* = 0.191<br>*n* = 120	R= −0.091<br>*p* = 0.356<br>*n* = 118	R = −0.013<br>*p* = 0.874<br>*n* = 107		
	**Burns patients**				
	**D1**	**D3**	**D7**	**D14**	**D28**
**Age**	R = 0.081<br>*p* = 0.567<br>*n* = 93	R = 0.146<br>*p* = 0.436<br>*n* = 84	R = −0.006<br>*p* = 0.980<br>*n* =74	R = 0.015<br>*p* = 0.980<br>*n* = 66	**R = 0.331**<br>***p* = 0.010**<br>***n* = 54**
**BMI**	R = 0.108<br>*p* = 0.473<br>*n* = 89	R = 0.006<br>*p* = 0.981<br>*n* = 81	R = −0.139<br>*p* = 0.436<br>*n* = 71	R = 0.119<br>*p* = 0.436<br>*n* = 63	R = 0.120<br>*p* = 0.436<br>*n* = 52

Table S2. Characteristics of trauma patients that did or not develop post-injury multiple organ dysfunction syndrome (MODS). Significant difference between groups are indicated in bold font. GCS, Glasgow Coma Scale; ICU, Intensive Care Unit; ISS, Injury Severity Score; Pen, Penetrating; RTC, Road Traffic Collison.

	**≤1 h**			**4–12 h**			**48–72 h**		
	**MODS (*n* = 52)**	**No MODS (*n* = 57)**	***P***	**MODS (*n* = 54)**	**No MODS (*n* = 55)**	***P***	**MODS (*n* = 57)**	**No MODS (*n* = 49)**	***P***
Age, years (range)	44 (18–90)	38 (18–81)	n.s	43 (18–90)	41 (18–95)	n.s	44 (18–90)	39 (18–95)	n.s
Gender, Male, *n(%)* Female, *n(%)*	46 (88.5) 6 (11.5)	47 (82.5) 10 (17.5)	n.s	48 (88.9) 6 (11.1)	46 (83.6) 9 (16.4)	n.s	51 (89.5) 6 (10.5)	42 (85.7) 7 (14.3)	n.s
ISS (range)	30 (9–66)	18 (9–50)	**<0.0001**	31 (9–66)	18 (9–50)	**<0.0001**	31 (9–66)	19 (9–50)	**<0.0001**
GCS (range)	7 (3–15)	13 (3–15)	**<0.0001**	7 (3–15)	13 (3–15)	**<0.0001**	7 (3–15)	14 (3–15)	**<0.0001**
Time to pre-hospital blood sample	43 (13–60)	42 (18–60)	n.s	42 (13–60)	42 (18–60)	n.s	42 (13–60)	41 (18–60)	n.s
Mechanism of Injury									
RTC, *n (%)*	34 (65.4)	22 (38.6)	**0.005**	37 (68.5)	25 (45.5)	**0.015**	38 (66.7)	23 (46.9)	**0.040**
Fall/Pen, *n (%)*	12 (23.1)	12 (21)	n.s	11 (20.4)	10 (18.2)	n.s	12 (21.1)	9 (18.4)	n.s
Assault/Pen, *n (%)*	5 (9.6)	5 (8.8)	n.s	5 (9.3)	4 (7.3)	n.s	5 (8.8)	4 (8.2)	n.s
Blunt/Pen, *n (%)*	1 (1.9)	18 (7)	**<0.0001**	1 (1.9)	16 (29.1)	**<0.0001**	2 (3.5)	13 (26.5)	**0.001**
ICU free days (range)	12 (0–30)	26 (0–30)	**<0.0001**	11 (0–30)	27 (9–30)	**<0.0001**	12 (0–30)	27 (0–30)	**<0.0001**
Hospital free days (range)	4 (0–22)	16 (0–28)	**<0.0001**	3 (0–22)	16 (0–28)	**<0.0001**	4 (0–22)	16 (0–28)	**<0.0001**
